# Whitefly-Transmitted Viruses of Cucurbits in the Southern United States

**DOI:** 10.3390/v15112278

**Published:** 2023-11-20

**Authors:** Ragunathan Devendran, Saritha Raman Kavalappara, Alvin M. Simmons, Sudeep Bag

**Affiliations:** 1 Department of Plant Pathology, University of Georgia, Tifton, GA 31793, USA; 2 U.S. Vegetable Laboratory, Agricultural Research Service, United States Department of Agriculture, Charleston, SC 29414, USA

**Keywords:** cucurbits, whitefly, viruses, begomovirus, crinivirus, ipomovirus, diagnosis, management

## Abstract

Cucurbits are economically important crops that are widely cultivated in many parts of the world, including the southern US. In recent years, higher temperatures have favored the rapid build-up of whiteflies in the fall-grown cucurbits in this region. As a result, whitefly-transmitted viruses (WTVs) have severely impacted the marketable yield of cucurbits. In this review, we discuss three major groups of WTVs negatively impacting cucurbit cultivation in the southern US, including begomoviruses, criniviruses, and ipomoviruses. Here, we discuss the available information on the biology, epidemiology and advances made toward detecting and managing these viruses, including sources of resistance and cultural practices.

## 1. Introduction

Cucurbits are important crops that are consumed as fruits or vegetables. They belong to the family *Cucurbitaceae* and comprise 965 species in 95 genera [1]. They are an excellent source of dietary fibers, vitamins, and essential minerals such as zinc, magnesium, and iron. Cucurbit seeds are also a good source of essential fatty acids. Many parts of cucurbits were used in traditional medicine in India, China, and Africa for treating various ailments [2]. Cucurbits are cultivated in more than 100 nations with 12 countries contributing to more than 70% of the total global yield [3]. The US is among the top cucurbit-growing nations, and has an estimated field production of nearly 109 million metric tons on about 229,000 hectares, and contributes to an economic value of approximately USD 1.43 billion [4]. Economically important cucurbit grown in the region include cantaloupe (*Cucumis melo* var. cantalupensis Naudin), cucumber (*C. sativus* L.), honeydew (*C. melo* L. (Inodorus Group) ‘Honey Dew’), muskmelon (*C. melo*), pumpkin (*Cucurbita* spp.), yellow squash (*C. pepo*), watermelon (*Citrullus lanatus*), and zucchini (*Cucurbita pepo* L.) [1].

Cucurbits can be infected by many pathogens including at least 59 different species of plant viruses from different genera [5]. In recent years, the production of cucurbits has been challenged globally due to the impact of whiteflies (notably *Bemisia tabaci*) (Hemiptera: Aleyrodidae) and the viruses that they transmit. In the states of Georgia and Florida, cucurbit production is heavily affected by these viruses and there are sporadic reports of incidence from other states in the Southern US. During the fall of 2015-2017, whitefly-transmitted diseases were responsible for 35% of crop losses in squash in Georgia [6]. Extensive yield losses were reported in Florida as well due to a tremendous population increase of *B. tabaci* that was complemented with virus incidence [7]. This review focuses on viruses infecting cucurbits in the Southern US, emphasizing Begomoviruses, Criniviruses, and Ipomoviruses transmitted by whiteflies.

## 2. DNA Viruses

### 2.1. Cucurbit Leaf Crumple Virus

Cucurbit leaf crumple virus (CuLCrV) is the most prominent DNA virus infecting cucurbits in the Southeastern US. CuLCrV was discovered in California during the fall of 1998 on volunteer watermelon in the Imperial Valley [8]. The virus was also found infecting pumpkin, muskmelon, and honeydew melon in Arizona and Texas around the same time [9,10]. Symptoms on squash included crumpled, curled and thickened leaves with disease incidence ranging from 35% to 95% [11]. CuLCrV belongs to the genus *Begomovirus* within the family *Geminiviridae* [10,12]. Generally, begomoviruses possess circular ssDNA genomes ranging from 2.5 to 2.7 kb [12]. The genome of CuLCrV is similar to other bipartite begomoviruses, and phylogenetic analysis placed the virus in the squash leaf curl virus (SLCV) cluster of New World bipartite begomoviruses [10]. 

**Figure 1:**
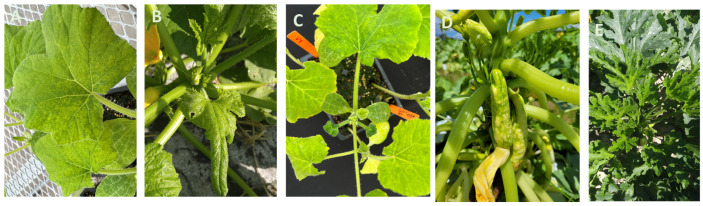
Symptoms caused by cucurbit leaf crumple virus (CuLCrV). Squash is the most affected crop by CuLCrV (**A**–**D**). Symptoms begin as diffuse yellow spots (**A**) and progress to crumpling (**B**,**C**) and stunting (**C**) in severe cases. The fruits on infected squash display green streaks and bumps (**D**). Symptoms of CuLCrV in zucchini (**E**) are milder than in squash and include mild chlorosis and crumpling. Photo Credit: S.R.K. and S.B.

In the southeastern US, CuLCrV was first reported on yellow straightneck and zucchini squash (*Cucurbita pepo* L.) in Florida in 2009 [13]. This was also the first time a begomovirus infection was reported on cucurbits in Florida and in the southeastern US. In Georgia, the natural incidence of CuLCrV is observed on all cucurbits grown in the fall including cantaloupe, cucumber, squash, and zucchini [14]. However, squash is the most affected crop with infection on 85% to 90% of samples tested and also suffered the most severe symptoms among all cucurbits [14]. Symptoms of CuLCrV on zucchini included green mosaic mottling and crumpling with the virus detected on 87% of samples tested. CuLCrV incidence was lower on cantaloupe (29–53%) and cucumber (53–69%) when compared to squash in the field [14]. Typical symptoms of leaf crumpling caused by CuLCrV on squash were also not observed on cucumber and cantaloupe. Under greenhouse conditions, symptoms in squash can be observed on the topmost leaves as early as 10 days post-inoculation when young seedlings are inoculated. The earliest symptoms include pale yellow spots on the leaves (Figure 1A). Newly emerging leaves become crumpled, curled and thickened (Figure 1B). Severely infected plants become very stunted (Figure 1C). Fruits on infected plants have green streaks and appear bumpy (Figure 1D). Leaf crumpling, the typical symptoms caused by CuLCrV on squash, was not observed on cucumber and cantaloupe. Apart from being a serious problem in Georgia and Florida, CuLCrV has also been identified in South Carolina [15] although heavy losses have not been reported.

CuLCrV is transmitted by the whitefly *B. tabaci* Gennadius (Middle East-Asia Minor 1 [MEAM1], formerly called biotype B) in a persistent and circulative manner [16,17]. CuLCrV can be transmitted transovarially as well as through mating by *B. tabaci*, with low frequency in each case. However, the rate of transovarial transmission was demonstrated to be low at only 3.93% in nymphs and 3.09% in adults [16]. Additionally, the recipient adults that acquired CuLCrV transovarially and via mating were not able to transmit the virus to squash plants suggesting that transovarial and mating CuLCrV transmission might not contribute to CuLCrV epidemics [16]. Seed transmission has recently been reported on begomoviruses [18,19,20], but is not known for CuLCrV. However, this is a very important aspect of CuLCrV transmission that needs to be studied.

### 2.2. Squash Leaf Curl Virus (SLCV) 

Squash leaf curl virus (SLCV) was the first begomovirus infecting cucurbits identified in the USA. It was recorded on squash exhibiting leaf curl and stunted growth symptoms in the early 1980s from California [21]. Characteristic symptoms in squash, melon, and watermelon include leaf curling, foliar mottling or mosaic with curling or stunting, blistering, and fruit deformation [5]. On pumpkin, infected plants display green or yellow mottle and mild foliar mosaic patterns with chlorotic spots [22]. SLCV was also found infecting watermelons in Texas [23] and Arizona [24]. There have not been any reports of widespread damage to cucurbits due to this virus after the initial documentation.

### 2.3. Watermelon Chlorotic Stunt Virus (WmCSV)

WmCSV is an Old World begomovirus initially reported from Yemen infecting watermelon plants in 1986, [25], and it is widespread in several regions of the Middle East. In the New World, it was first reported from Sonora, Mexico, infecting watermelon plants during the summer of 2012 [26]. In cucurbits, the symptoms include stunting, vein yellowing, and the appearance of chlorotic patches on leaves [27]. Under laboratory conditions, it could infect cucurbits such as melon, squash, watermelon and non-cucurbits such as *Nicotiana benthamiana* and tomato [27]. A few years after its emergence, it was detected in cactus (Cactaceae family) plants of Arizona, USA, along with SLCV, indicating a possible spillover from an agricultural area to the natural vegetation [28]. The identification of WmCSV in the USA and Mexico indicates that this virus could be broadly distributed in the New World [27].

## 3. RNA Viruses

### 3.1. Criniviruses

Criniviruses belong to the genus *Crinivirus* in the family *Closteroviridae* [29] and pose a serious threat to cucurbit production worldwide [30]. Criniviruses have non-enveloped filamentous particles and the genome consists of bipartite ssRNAs in most of the members [29]. In the southeastern US, two criniviruses have been reported to infect cucurbits, including cucurbit chlorotic yellows virus (CCYV) and cucurbit yellow stunting disorder virus (CYSDV). The genomes of CCYV and CYSDV are very similar with the only exception being the presence of putative proteins p5 and p25 in place of p6 protein in CCYV towards the 3′ end of RNA 1. CCYV and CYSDV are transmitted by cryptic species of *B. tabaci,* MEAM1 and Mediterranean (MED, formerly Q) [31,32,33]. The mode of transmission is semi-persistent which means that the whiteflies can acquire and transmit the virus in a short period of time [34].

#### 3.1.1. Cucurbit Yellow Stunting Disorder Virus (CYSDV)

**Figure 2:**
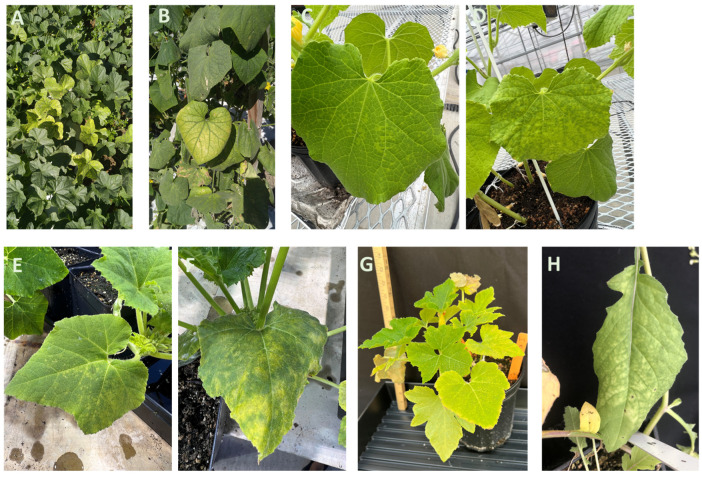
Symptoms of criniviruses on cucurbits. Chlorosis on the crown region of cantaloupe (**A**) and cucumber (**B**) in commercial fields due to mixed infection of cucurbit chlorotic yellows virus (CCYV) and cucurbit yellow stunting disorder virus (CYSDV). Initial symptoms of CCYV infection as yellow spots on cucumber (**C**,**D**) and squash (**E**,**F**). Severe chlorosis is on the lower leaves of squash (**G**) and interveinal chlorosis is on wild radish infected with CCYV (**H**). Photo Credit: S.R.K. and S.B.

CYSDV was discovered in the United Arab Emirates in 1982 on melons [35] and is now prevalent in many tropical and subtropical cucurbit-growing regions of the world [35,36,37]. In North America, CYSDV was observed in 1999 from field and greenhouse-grown *Cucumis melo* in Texas [38], followed by California and Arizona [24], and it became widespread in the western parts of the Sonoran Desert in Arizona and Sonora, Mexico [39]. Soon thereafter, it was found infecting squash in Florida (*C. pepo* L.) [40]. In Georgia, the virus was first identified as infecting cucumber, cantaloupe and yellow squash in 2016 [41]. The symptoms of CYSDV initially appear as a yellow-green chlorotic mottle, which develops into interveinal chlorosis later [36]. Yellowing or interveinal chlorosis are more pronounced on older leaves [36] (Figure 2A,B). Early infection of cucumber, melon, and watermelon plants by CYSDV results in reduced yields. Fruit number and weight are reduced in infected plants [42]. In a survey conducted in 2019 and 2020 in Georgia, natural infection of CYSDV was found on cantaloupe, cucumber, yellow squash, and zucchini squash with the highest incidence on cantaloupe and cucumber [14]. CYSDV is often detected as co-infection with other viruses including CuLCrV and CCYV which are also transmitted by the same vector [14]. CYSDV was also found recently infecting watermelon in South Carolina [43] and commercial fields in Alabama [44].

#### 3.1.2. Cucurbit Chlorotic Yellows Virus (CCYV)

CCYV was first reported in 2004 from Japan where it caused heavy losses in melon production [45]. CCYV is now present in Taiwan [46], China [47], India [48], Sri Lanka [49], Philipines [50], Sudan [51], Greece [52], Spain [53], Iran [54], and Lebanon [55]. In the US, it is an important emerging virus in cucurbit crops such as melons and watermelons [56]. CCYV was introduced in the southwestern US in 2014 [57]. The symptoms of CCYV are nearly indistinguishable from that produced by CYSDV. Symptoms start as chlorotic spots with diffuse margins which later coalesce and develop into interveinal chlorosis. On an infected plant, older leaves appear brittle with interveinal chlorotic spots, while younger leaves are also chlorotic with yellowing between the veins (Figure 2C–F) [58]. Early infection of cucumber, melon, and watermelon plants by CCYV results in reduced yields and reduced sugar content in melons [59]. In the Southeast, CCYV was first detected in Georgia [60] followed by Florida [61], and Alabama [62]. Under natural conditions, CCYV causes diseases in cantaloupe (*Cucumis melo)*, cucumber (*C. sativus*)*,* and watermelon (*Citrullus lanatus)* [33]. Under experimental conditions, CCYV could cause infection in other cucurbit hosts such as *Luffa cylindrica* [33] and non-cucurbit hosts such as *Beta vulgaris* (beet)*, Chenopodium amaranticolor, C. quinoa, Datura stramonium* (datura)*, Spinacia oleracea* (spinach)*, Lactuca sativa* (lettuce)*,* and *Nicotiana benthamiana* [33]. Mixed infections of CCYV with CYSDV and CuLCrV are common in the southeastern US. CCYV isolates from Georgia clustered with the Asian isolates of the virus [14]. Symptoms of CYSDV and CCYV infection may also be confused with those caused by various nutritional deficiencies or by those associated with other diseases, such as cucurbit yellow vine disease and downy mildew.

### 3.2. Ipomovirus 

**Figure 3:**
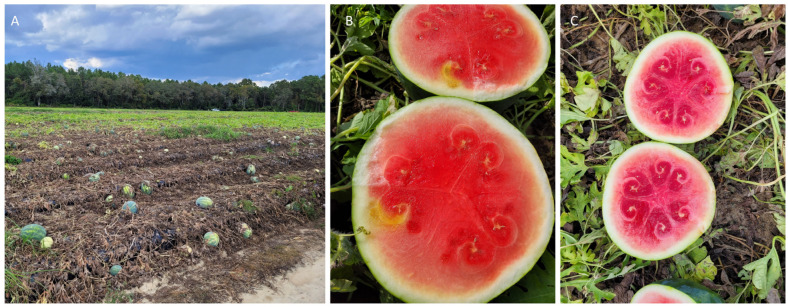
Symptoms of watermelon vine decline disease caused by squash vein yellow virus. The vine declined at the time of harvest (**A**), with discolored blotch and necrotic symptoms on the rind (**B**) and dark red color on the flesh (**C**) on a commercial watermelon field in Georgia during the Fall of 2023. Photo Credit: S.B. and S.R.K.

In 2003, a previously unknown virus was detected in squash in Florida [63]. The host range, mode of transmission and genome sequence indicated that this virus is a previously undescribed *Ipomovirus* and the name *Squash vein yellowing virus* (SqVYV) was proposed [63]. *Ipomoviruses* are whitefly-transmitted plant viruses in the family *Potyviridae* and possess filamentous flexuous virions [64,65]. The genome organization of SqVYV is similar to other potyvirids, but without a Helper Component-Proteinase (HC-Pro) that aids aphid transmission of potyviruses [64]. Unlike other potyviruses, SqVYV is transmitted by *B. tabaci* MEAM1 in a semi-persistent manner [66]. In 2005, this virus was linked to watermelon vine decline (WVD) in Florida and caused severe monetary losses to watermelon growers in South Florida [67]. The symptoms of WVD start with yellowing of the foliage followed by browning and the collapse of the entire vine within weeks of the first symptoms (Figure 3A). The symptoms appear as the fruits approach harvestable size. The fruits have discolored blotches on the rinds (Figure 3B) and the flesh is often too red (Figure 3C) with an off taste [63,68]. 

Under experimental conditions, SqVYV infects a wide variety of cucurbits such as pumpkin, tropical pumpkin, squash, and luffa. The virus is devastating mainly due to its ability to cause vine decline and fruit rot in watermelons. On other cucurbits, such as pumpkin, squashes, and in luffa, SqVYV develops vein yellowing in inoculated and systemic leaves, while in cantaloupe and cucumber vein yellowing was transient in systemic leaves immediately above the inoculated leaves. SqVYV also infects cucurbit weeds such as balsam apple (*Momordica balsamina*) and smellmelon (*Cucumis melo*) under natural conditions and creeping cucumber (*Melothria pendula*) under experimental conditions [69]. Squash vein yellowing virus (SqVYV) was isolated from ivy gourd (*Coccinia grandis*) [69]. SqVYV failed to infect plants other than cucurbits tested [43]. In balsam apple, vein yellowing was observed throughout the plant [69]. SqVYV is now widely distributed in watermelon, squash and cucurbit weeds in southwest and west central Florida [70], and has also recently been reported from South Carolina [68]. SqVYV was reported from Georgia in 2011 [71] but not detected again until recently. In the fall of 2023, SqVYV was detected in a commercial field in Georgia causing vine decline in 70-80% of the crop (Figure 3A–C). 

## 4. Whiteflies, the Leading Carrier 

Globally, whiteflies (Hemiptera:Aleyrodidae) have long been economically important agricultural pests. Whiteflies transmit more than 300 known plant pathogenic viruses to more than 1000 known plant species across the world [72,73]. The most widely known species of whiteflies are *B. tabaci* (sweet potato whitefly) and *Trialeurodes vaporariorum* Westwood (greenhouse whitefly) [73,74]. These two species are among the most destructive insect pests of agricultural crops, vegetables, and ornamental plants in the southern US [73]. Specifically, *B. tabaci* is a major threat to vegetable production in the southern US. *Bemisia tabaci* (MEAM1) was initially introduced in the US around 1985 and has since rapidly spread across the southern US [75,76]. Following the introduction of this whitefly to the US, it has become a primary vector responsible for the transmission of several virus species. Besides virus transmission, whiteflies can also cause severe injury through feeding and secreting honeydew. The preference of whiteflies for cucurbits over other crops in the agrosystem could explain why they are more vulnerable to whitefly-transmitted viruses (WTVs). For example, whiteflies MED and MEAM1 species show a preference for cucumber over pepper and tomato for colonizing and ovipositing [77,78,79].

## 5. Other Emerging Viruses of Cucurbits

The development of high-throughput sequencing (HTS) and bioinformatics has accelerated the discovery of novel viruses. The use of high-throughput sequencing facilitated in the discovery of other plant viruses infections recently in Georgia. Because very little information is available about these viruses, they are mentioned in this article.

### 5.1. Watermelon Crinkle Leaf-Associated Virus 1 and Watermelon Crinkle Leaf-Associated Virus 2 

Watermelon crinkle leaf-associated virus 1 (WCLaV-1) and watermelon crinkle leaf-associated virus 2 (WCLaV-2) are rather recently discovered viruses and not much is yet known about them. Both viruses were discovered in China by HTS on watermelon displaying virus-like symptoms, including leaf crinkling, mosaic, and bunchy top in samples collected in 2015 and 2016 during a field survey [80]. WCLaV-1 and WCLaV-2 were believed to have multipartite genomes consisting of three RNA molecules of ~6.8, 1.4, and 1.3 kb when they were discovered. These two viruses were placed in a novel clade within the family *Phenuiviridae* in the order *Bunyavirales* [80]. However, further studies revealed that the genome of WCLaV-1 and WCLaV-2 is indeed bipartite consisting of a negative-sense RNA1, encoding the RNA-dependent RNA polymerase, and an ambisense RNA2, encoding the putative movement (MP) and nucleocapsid (NP) proteins [81]. Based on these features and phylogenetic reconstructions, WCLaV-1 and WCLaV-2 has been provisionally assigned to the genus Coguvirus (family *Phenuiviridae*) [81].

In 2021, these viruses were reported from Texas on watermelons displaying symptoms consisting of mild leaf crinkling and yellow mosaic patterns [82]. Thereafter, these viruses were also found infecting watermelons in Florida [83] and Georgia [84]. Apart from the USA, these viruses were recently reported in Australia [85] and Brazil [86].

WCLaV-1 infected watermelon plants displayed mosaic, crinkling, and bunching symptoms in Georgia, as described previously. The virus was detected in five counties within south Georgia, indicating its widespread prevalence in the state [87]. WCLaV-2, which was consistently found to be associated with WCLaV-1, was not identified in Georgia. So far, watermelon is the only known host of WCLaV-1 and WCLaV-2. Neither of these viruses was detected in cantaloupe grown at the same time as watermelon in Georgia by HTS and PCR. Further studies including biology, vector relations and economic significance of the virus need to be carried out starting with the standardization of a protocol for transmission. So far, WCLaV-1 can be transmitted mechanically to watermelon at only a very low frequency of 2–5% [80]. WCLaV-1 and WCLaV-2 infected watermelon also had very high levels of thrips population but the role of thrips or any other vector has not been investigated [80]. The nearly complete genome of an isolate of WCLaV-1 from Georgia (RNA1-OM751928 and RNA2-OM751930) assembled from small RNAs did not show any significant divergence from those reported in Brazil (100% identity with RNA 1-LC636070 and 99% with RNA 2-LC636069;) and China (99% for RNA 2-MW751424.1). In a recent survey conducted in Florida, WCLaV-1 was found to be the predominant virus in cucurbits, followed by CYSDV and WCLaV-2 [88].

### 5.2. Persistent Viruses

Unlike acute viruses, persistent viruses do not cause symptoms in infected plants. Hence, these viruses were previously called “cryptic” viruses [89]. For the same reason, they have been poorly studied although their existence has been known for a very long time. However, with metagenomic studies becoming more common, the abundance of persistent viruses has been revealed [90,91,92]. Three persistent viruses viz. *Cucumis melo* endornavirus (CmEV), *C. melo* amalgavirus (CmAV1), and *C. melo* cryptic virus (CmCV) were recently reported in Georgia in the spring of 2021. CmAV1 and CmEV were detetcted on watermelon and cantaloupe. CmCV was identified by HTS on watermelon; however, the virus could not be detected by RT-PCR in any of the field samples.

CmEV belongs to the genus *Endornavirus* and the family *Endornaviridae* while CmCV belongs to *Deltapartitivirus* of the family *Partitivirdae*, and CmAV1 is a virus member of the genus *Amalgavirus* of the family *Amalgaviridae.* Persistent plant viruses are not known to be transmitted horizontally by vectors [93]. However, they can be vertically transmitted at nearly 100% levels through both ova and pollen [94]. Persistent viruses lack any movement protein and do not move between plant cells but rather infect every cell and move by cell division and are transmitted through seeds [95].

## 6. Mixed Infections of Whitefly-Transmitted Viruses (WTVs) Are Common

Mixed infection refers to the existence of more than one virus at a given time in a host. Mixed infections have the potential to result in higher yield loss [96] and breakdown of field resistance in crops [97]. Mixed infections of WTVs in cucurbit crops are common in Florida [69,88,98], Georgia [14], and South Carolina [43]. In some cases, mixed infection of viruses is known to alter the plant phenotype severely compared with single infections. Such alterations in phenotypic traits can further increase the attractiveness of plants to the vector than singly-infected plants [99]. For example, CuLCrV and CYSDV in mixed infections caused more severe symptoms on squash than any of these viruses individually [17].

The accumulation of criniviruses, CCYV and CYSDV was significantly decreased compared to single virus-infected plants under greenhouse [100] and field-collected samples [101]. In cucumber, the accumulation of both CCYV and CYSDV and subsequent transmission efficiency of each of these viruses by whiteflies were significantly decreased during mixed infections compared to those during single infections. However, their simultaneous transmission (both viruses transmitted together) efficiency was significantly higher [100]. Crop-dependent preferential accumulation and transmission of one of the viruses among CCYV, CYSDV, and CuLCrV over others during mixed infections was observed. CYSDV accumulated in significantly lower amounts in mixed (CuLCrV and CYSDV)-infected squash plants than in CYSDV-infected plants. As a result, whiteflies acquired similar levels of CuLCrV, but reduced levels of CYSDV from mixed-infected squash plants compared to plants infected with only any one of these viruses [17]. However, it is unclear if the reduced accumulation of CYSDV in mixed-infected plants and reduced acquisition by whiteflies thereafter would suppress whitefly-mediated inoculation of CYSDV compared with acquisition and inoculation from CYSDV-infected plants. During surveys in 2019 and 2020, squash samples infected with CuLCrV were larger than those infected with CCYV and CYSDV [14]. On the other hand, crinivirus infections, of either CCYV or CYSDV were more prominent on cucumber and cantaloupe. These results indicate that mixed infection of viruses in host plants and acquisition of multiple viruses by the vector could have implications for virus accumulation, virus acquisition, vector preference, and epidemics that sometimes are different from single-virus infection or acquisition. Therefore, it is possible that virus accumulation differences in host plants (and subsequently in vectors) following virus-virus interactions associated with mixed infection could play a crucial role in influencing the epidemics of component viruses.

## 7. Alternate Hosts as Potential Reservoirs

CuLCrV virus has a wide host range which includes squash, watermelon, cantaloupe, and many gourds, whereas honeydew melon, crenshaw melons, and casaba melons were least susceptible [10]. Heavy incidence of CuLCrV is also reported on *Phaseolus vulgaris* (snap beans) in Florida [13] and Georgia [102]. Not much work has been reported on weeds that harbor CuLCrV in the field.

CYSDV infects both cucurbits as well as non-cucurbit crops. The main cucurbit hosts in the Southeast includes cucumber, cantaloupe, squash, and watermelon. In California, some non-cucurbit crops such as *Lactuca sativa* (lettuce), snap bean, *Medicago sativa* (alfalfa), and some weed species such as *Solanum elaeagnifolium* (silverleaf nightshade), *Malva neglecta* (common mallow), *Sisymbrium irio* (London rocket), *Physalis wrightii* (Wright’s groundcherry), and *Sida hederacea* (alkali mallow) were found to harbor CYSDV [103]. These crops and weeds are also abundant in the southeastern US and could serve as a potential reservoirs for CYSDV. Similarly, pigweed (*Amaranthus spp*.) is a potential reservoir for CYSDV in Florida [104] while wild radish (*Raphanus raphanistrum* L.) is potential reservoir for CCYV in Georgia [105]. *Momordica charantia* is a potential reservoir host of SqVYV [70]. Common cucurbit weeds like balsam apple and smellmelon are natural hosts of SqVYV, and creeping cucumber is an experimental host [69]. 

Unlike crop hosts, weeds can survive even during non-crop seasons. Weeds grown during the summer support the multiplication of whiteflies while winter weeds support continuity of whitefly population. Whitefly populations gain a foothold when weather warms and they multiply quickly during the summer. Identifying the appropriate weeds that support whiteflies and the WTVs and their timely removal are essential to break the continued life cycle of both whiteflies and WTVs [63]. 

## 8. Diagnosis

The success of virus disease detection and management depends upon diagnostics methods and early detection of viruses. Characteristic symptoms induced by viruses on diseased plants is the preliminary step in diagnosis, but this is nearly impossible due to the mixed infections of more than one virus of the same or different genus on a diseased plant at a time. Serological diagnostic tools are not available for any of the whitefly-transmitted viruses present in the Southeast US. It would be much simpler and lower cost if such a technique were available to screen large number of samples such as those required for epidemiological studies. 

CuLCrV concentrations are higher on the upper (young) leaves while that of criniviruses, CCYV and CYSDV are higher on the lower (old) leaves. Hence, samples for the detection of CuLCrV should be taken from upper leaves while those for CCYV and CYSDV should be taken near crown of the plants. SqVYV is unevenly distributed in its hosts and often appears to have a low titer in many tested plants [63]. Hence, the diagnosis of this virus can be difficult. Typical potyvirus inclusions are not always found in leaf strips and leaf dips, and thus, their analysis using electron microscope can be negative. Currently, no antiserum is available for this virus. The best test is a RT-PCR or a nested RT-PCR assay using primers based on the sequence of the capsid protein gene of SqVYV. Plant samples for detection of SqVYV should be taken from the crown of the infected plant [63]. Mechanical inoculation of watermelons with samples can also be useful for detecting SqVYV as death of the inoculated plant is a diagnostic symptom [68].

A simple one-step multiplex RT-PCR system has recently been developed for the simultaneous detection of cucurbit leaf crumple virus, cucurbit yellow stunting disorder virus, squash vein yellowing virus, and cucurbit chlorotic yellows virus [63]. This assay has the potential to reduce cost, time and labor for the diagnosis of a large number of samples. Another multiplex PCR assay to detect WTVs present in California including CuLCrV, CCYV, and CYSDV has been developed [63].

Loop-mediated isothermal amplification (LAMP) is an isothermal technique that does not require sophisticated instruments like thermal cyclers. A LAMP assay was developed for simple, rapid and efficient detection of CuLCrV. The sensitivity assay demonstrated that the LAMP reaction was more sensitive than conventional PCR, but less sensitive than qPCR. However, it was simpler and faster than PCR and qPCR. Furthermore, this assay was able to detect CuLCrV in mixed virus infections [63]. Recombinase polymerase amplification (RPA) assays are also being developed for detection of these viruses at the University of Florida. 

HTS combined with subsequent application of bioinformatics for the detection and identification of both known and novel plant viruses and other pathogens has been proven to be successful with different sequencing platforms using nucleic acid preparation as the starting material [106]. Moreover, HTS does not require previous knowledge of viral sequences. The sensitivity of HTS was ten times higher than RT-qPCR [107]. Compared to other techniques, this technique is time-consuming, and expensive so it cannot be employed for regular diagnosis. HTS also revealed a significant percentage of mixed infection of viruses among the samples tested [14].

## 9. Disease Management and Control

No single management tactic is effective enough to suppress whiteflies and reduce the transmission of viruses in Georgia and other southeastern states. Several management tactics aimed at reducing the impacts of *B. tabaci* MEAM1 and whitefly-transmitted viruses were evaluated in Georgia. Among them, insect exclusion netting (IEN) significantly reduces whiteflies and virus incidence on squash seedlings in the greenhouse [108]. In the field, lower whitefly abundance and reduced virus symptom severity, were observed in plots with UV-reflective mulch when compared to white plastic or live mulch. Overall, field plots with row covers and those with UV-reflective mulch consistently produced the greatest marketable yields [108]. Growers can reduce whitefly and virus pressure by combining these cultural tactics, and selecting insecticides to preserve yields in squash production in the southeastern US [108]. Insecticides and silver plastic mulch have been effective in managing whiteflies and watermelon vine decline in Florida [109,110]. Results from studies conducted on squashes cultivated both in experimental plots and grower’s field revealed that whitefly infestation and the virus transmitted by them generally initiated along the edges, which later spread to plants within the rows [111]. In such a scenario, phytosanitary techniques or planting cover crops along the edges of fields could play an important role in mitigating yield losses caused by whitefly-transmitted viruses.

Using cultivars with resistance to either vectors or the viruses transmitted by them is an economical and effective way to avoid yield loss. Currently, no cucurbit cultivars with resistance to CuLCrV, CYSDV, or CCYV are available commercially, but efforts are underway to identify sources of resistance [112,113]. A melon breeding line PI 236355 is completely resistant while MR-1, PI 124112, PI 179901, PI 234607, PI 313970 and PI 414723 have partial resistance against CuLCrV. A single recessive gene *culcrv*, controls the resistance in PI 313970, a *C. melo* accession and likely in the other resistant accessions [113]. 

Resistance to CCYV was reported in a snap melon (Momordica group) (accession: JP 138332), originating from India [114]. This line accumulated relatively lower CCYV titer compared to other melon accessions tested. A single recessive QTL located on chromosome 1 was found to be associated with resistance in this line [115]. 

Regarding CYSDV, resistance to cucurbit yellow stunting disorder virus was first reported in the accessions TGR1551 (C-105, PI 482420) and TGR-1937 (PI 482431) [42]. Characteristics of resistance include delayed and mild symptoms and were reported in a few accessions under natural infection conditions in the United Arab Emirates (Jupiter, Muskotaly, PI 403994) [116] and in Spain [42]. The resistance in TGR1551 was initially reported to be controlled by a single dominant gene called *Cys* [42], but re-evaluations showed an inheritance pattern consistent with that of a recessive gene [117]. Two QTLs are associated with resistance to CYSDV in TGR-1551 located near chromosome 5 and the interval between the two loci is mapped to be approximately 700 kb [118]. Several accessions of Indian origin, most notably PI 313970, which also has CuLCrV resistance showed resistance to the California and Arizona CYSDV strains of CYSDV [119]. Resistance in this accession was also reported as monogenic recessive [120] and likely allelic to the resistance in TGR-1551 [121]. Screening of germplasm under natural incidence in Georgia and Florida (USA) identified several accessions that are potential sources of resistance to CuLCrV, CYSDV, and whiteflies [122]. Several accessions of cucumber including PI 211589, PI 605923, and Ames 13334, developed less severe symptoms than the susceptible accessions, while PIs 177364, 279807, 29342, and NSL 5476 performed better with low disease pressure under field conditions [123]. Several watermelon accessions with moderate resistance to SqVYV have been identified [124]. PI 392291 is resistant to vine decline caused by SqVYV and could serve as an important source of resistance [125]. 

## 10. Conclusions

Over the years, the production of cucurbits in the US has experienced challenges due to whiteflies and whitefly-transmitted viruses. In recent years, along with a higher incidence of whiteflies there has been an increase in the incidence of plant virus diseases they transmit. Mixed infections of two or more viruses are common adding to severity of impact in many cases. Efforts are underway to mitigate the grower’s losses and to increase profitability and sustainability. Ongoing research on identifying the resistance sources of whiteflies and the viruses they transmit, identifying overwintering hosts, and developing integrated pest and disease management practices is promisingMolecular understanding of the aspects of host-virus interaction, synergism in cucurbits, whitefly-host-virus interactions etc., is also needed to develop novel strategies of disease management and crop protection. 

## Data Availability

Not applicable.
